# China CO_2_ emission accounts 1997–2015

**DOI:** 10.1038/sdata.2017.201

**Published:** 2018-01-16

**Authors:** Yuli Shan, Dabo Guan, Heran Zheng, Jiamin Ou, Yuan Li, Jing Meng, Zhifu Mi, Zhu Liu, Qiang Zhang

**Affiliations:** 1 Water Security Research Centre, Tyndall Centre for Climate Change Research, School of International Development, University of East Anglia, Norwich NR4 7TJ, UK; 2 Department of Earth System Sciences, Tsinghua University, Beijing 100080, China; 3 Department of Politics and International Studies, University of Cambridge, Cambridge CB3 9DT, UK; 4 Bartlett School of Construction and Project Management, University College London, London WC1E 7HB, UK

**Keywords:** Climate-change policy, Environmental economics, Environmental chemistry

## Abstract

China is the world’s top energy consumer and CO_2_ emitter, accounting for 30% of global emissions. Compiling an accurate accounting of China’s CO_2_ emissions is the first step in implementing reduction policies. However, no annual, officially published emissions data exist for China. The current emissions estimated by academic institutes and scholars exhibit great discrepancies. The gap between the different emissions estimates is approximately equal to the total emissions of the Russian Federation (the 4th highest emitter globally) in 2011. In this study, we constructed the time-series of CO_2_ emission inventories for China and its 30 provinces. We followed the Intergovernmental Panel on Climate Change (IPCC) emissions accounting method with a territorial administrative scope. The inventories include energy-related emissions (17 fossil fuels in 47 sectors) and process-related emissions (cement production). The first version of our dataset presents emission inventories from 1997 to 2015. We will update the dataset annually. The uniformly formatted emission inventories provide data support for further emission-related research as well as emissions reduction policy-making in China.

## Background & Summary

With lifestyle changes and rapid economic growth in China, the CO_2_ emissions in China have increased rapidly. The CO_2_ emissions from fossil fuel combustion (energy-related emissions) and cement production (process-related emissions) in China rose steadily and slowly in the pre-WTO era (1980–2002). These emissions increased from 1,467 to 3,694 million tonnes during this period^1^, a rate of 8% per year. After China joined the WTO in 2002, manufacturing in China quickly started to expand. Thus, China’s emissions also spiked. The annually averaged emissions rate increase from 2002 to 2007 reached 13%. This expansion led China to become the world’s top energy consumer and CO_2_ emitter^2^. Now, the human-induced CO_2_ emissions in China account for approximately 30% of global emissions^3^. Consequently, China is playing an important role in global emissions reduction and climate change mitigation. The Chinese government has promised that its CO_2_ emissions will peak by 2030^4^ and that it will achieve a 60%-65% reduction in its emission intensity (per GDP CO_2_ emissions) by 2030 compared with its 2005 level^5^.

An accurate accounting of China’s CO_2_ emissions is the first step in achieving emissions reductions. However, the CO_2_ emissions accounts for China have not been well documented. There is no annual, officially published emission report in China. The Chinese government has only published national CO_2_ emission inventories for 1994^6^, 2005^7^, and 2012^8^. Scholars and research institutes have previously assumed the responsibility for calculating China’s CO_2_ emissions. The discrepancy between their estimations exceeded 1,770 million tonnes (20%) in 2011, which is approximately equal to the Russian Federation’s total emissions in 2011^9^. Considering that the Russian Federation was the 4th highest emitter in the world at that time^3^, the uncertainties in China’s emission accounts should not be underestimated. Compared with the three official CO_2_ emissions in China for 1994, 2005, and 2012, the estimates by international academic institutes have been relatively high. For example, in 2012, the Emission Database for Global Atmospheric Research (EDGAR) and Carbon Dioxide Information Analysis Centre (CDIAC) estimates were 10,057 and 10,020 million tonnes, respectively, which are 8% higher than the official estimate of China’s emissions (9,323 million tonnes). The primary reason is that nearly all the research institutes and scholars use the default emission factors recommended by IPCC, which are higher than China’s survey value^10^. The energy data quality is another reason for the limited veracity of China’s emission accounts^11^. Furthermore, all the existing datasets only present the national total CO_2_ emissions. There are scarcely any emission inventories constructed according to fossil fuel types and industrial sectors for China and its 30 provinces.

Considering the large uncertainties/data gaps in China and its provincial CO_2_ emission accounts, our first version of the dataset presents the CO_2_ emission inventories of China and its 30 provinces from 1997 to 2015. We also provide the national and provincial energy data used in the calculation for transparency and verifiability. We will update and publish the dataset annually. Our emissions are calculated based on the updated emission factors^10^ and most up to date energy consumption data^12^. The inventories are constructed in a uniform format, which includes emissions from 17 fossil fuels burned in 47 socioeconomic sectors (energy-related emissions) and those from the cement production industry (process-related emissions). The uniformly formatted time-series emission inventories can be utilized widely. These inventories can provide robust data support for further analysis of China’s environmental issues^13–17^ and emissions reduction policy-making^13^. The data can be downloaded freely from China Emission Accounts and Datasets (CEADs, www.ceads.net) and Figshre.

## Methods

**Figure 1:**
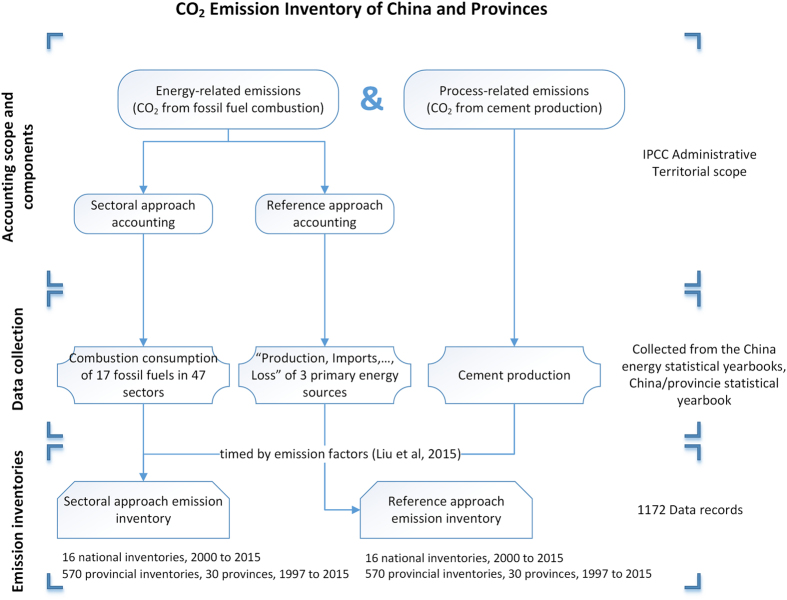
Diagram of CO_2_ emission inventory construction.

The CO_2_ emissions in this dataset were estimated in terms of the IPCC administrative territorial-based accounting scope. The administrative territorial emissions refer to emissions *‘taking place within national (including administered) territories and offshore areas over which the country has jurisdiction (page overview.5)’*^18^. The territorial-based emissions do not include emissions from international aviation or shipping^19^. The administrative territorial emissions can be used to evaluate the human-induced emissions by domestic production and resident activities directly within one region’s boundaries^20,21^. Our CO_2_ emission inventories were constructed in two parts: energy- and process-related (cement) CO_2_ emissions. The energy-related emissions can be calculated using two approaches: the sectoral and reference approaches. Figure 1 presents a diagram of the entire construction of our emission inventories.

### Energy-related sectoral approach emissions

The energy-related emissions refer to the CO_2_ emitted during fossil fuel combustion. According to the IPCC guidelines^22^, the sectoral approach emissions are calculated based on the fossil fuels’ sectoral combustion; see equation (1) below.

(1)CEij=ADij×NCVi×CCi×Oij

where *CE*_*ij*_ refers to the CO_2_ emissions from fossil fuel *i* burned in sector *j*; *AD*_*ij*_ represents the fossil fuel consumption by the corresponding fossil fuel types and sectors; *NCV*_*i*_ refers to the net caloric value, which is the heat value produced per physical unit of fossil fuel combustion; *CC*_*i*_ (carbon content) is the CO_2_ emissions per net caloric value produced by fossil fuel *i*; and *O*_*ij*_ is the oxygenation efficiency, which refers to the oxidation ratio during fossil fuel combustion.

The subscripts *i* (fossil fuel) and *j* (sector) correspond to those used in Table 1 and Table 2. There are 26 fossil fuels in China’s energy statistics systems, listed in the most recent energy balance table in the China energy statistical yearbook. We merged these fuels into 17 types due to the small consumption and similar quality of certain fuels to that of others, as shown in Table 1. Among the 17 fossil fuels, raw coal, crude oil, and natural gas are primary energy sources. The remaining 14 fuels are classified as secondary energy sources, which are extracted or processed from primary sources. The 47 sectors used in the energy statistical system are also consistent with those used in China’s national economic accounting^23^ (see Table 2). Due to all the administrative boundaries (at both the national and provincial scales) that span both urban and rural geographies in China, urban and rural households are listed separately in the multi-scale CO_2_ emission inventories.

Fossil fuels used as chemical raw materials (‘non-energy use’ in the Energy Balance Table), as well as the energy loss during transportation, were removed from the total fossil fuel consumption to avoid double counting. The non-burning fossil fuels input during energy conversion processes was also excluded as the processes involve little CO_2_ emissions. Taking the process of coal washing as an example, the carbon elements in raw coal are converted into cleaned coal and other washed coal during the process. The real CO_2_ emissions concentrated in the combustion of cleaned coal and other washed coal. Other similar processes include ‘coking’, ‘petroleum refineries’, ‘gas works’, ‘briquettes’. Only fossil fuels burnt during the transformation processes were taken into account for emission calculation, i.e., ‘thermal power’ and ‘heating supply’.

Emissions from electricity/heat generated within city boundaries were counted based on the energy input for power/heat generation (‘thermal power’ and ‘heating supply’) and were allocated to the electricity generation sector^24^. Our administrative territorial emission inventories excluded emissions from imported electricity and heat consumption from outside the nation/one province boundaries. We only focused on fossil fuels consumed within the nation/one province boundary.

The national sectoral fossil fuel consumption (*AD*_*ij*_) was collected from the Energy Statistical Yearbooks published officially by the National Bureau of Statistics of China^25^. China has officially revised its national energy statistics four times since 2000 (in 2004, 2005, 2009, and 2014’s China energy statistical yearbooks). Each revision has modified the energy balance sheets and sectoral energy consumption. For example, the total energy consumption of 2011 are modified from 3,480 to 3,870million tonnes of standard coal equivalent (in coal equivalent calculation) in 2014’s revision, enlarged by 11.2%. Our emission inventories were calculated based on the most up to date energy data published after 2014^25^.

For the provincial scale, the China Energy Statistical Yearbooks only publish each province’s energy balance table every year. We collected the total consumption of the 17 fossil fuels from the balance table and then used the provinces’ sectoral fossil fuel consumption to divide the total consumption. Most of the provinces’ sectoral fossil fuel consumption was collected from the provinces' corresponding statistical yearbooks. For certain provinces (Hebei, Jiangsu, Zhejiang, Shandong, Guangxi, Hainan, Sichuan, and Guizhou) that do not have the data in their yearbooks, we used the national economic census data from 2008^26^, which assumes the industry structure was stable during the intervening years.

Both the IPCC and National Development and Reform Commission of China (NDRC) have published default factors (*NCV*_*i*_, *CC*_*i*_) for China. Most of the current research uses the IPCC default value. According to our previous survey on China’s fossil fuel quality and cement process^10^, the IPCC default emission factors are approximately 40% higher than China’s survey value. In our datasets, we used the updated emission factors, see Table 1. As our previous study only reported the emission factors of three primary fossil fuels (i.e., raw coal, crude oil, and natural gas), we estimated the emissions factors of other 14 secondary fossil fuels by scaling them down according to the ratio of the updated primary fossil fuels’ emission factors to those of NDRC. We used the ratio of raw coal, crude oil to update emission factors of coal-related, oil-related fuels, respectively. For *O*_*ij*_, our datasets adopted different oxygenation efficiencies for the fossil fuels used in different sectors^27^, which represents the different combustion technology levels of the sectors (shown in Table 3 (available online only)).

We used MATLAB R2014a to construct the emission inventories with sectoral fossil fuel consumption and emission factors. We provided the code in the Supplementary Information. We also provided the formatted energy data of China and its provinces (energy inventories) in our datasets for additional data transparency and verifiability (see Data Citation 1, File ‘China national energy inventory, 2000–2015’ and File ‘China provincial energy inventory, 1997–2015’). Researchers will be able to use the MATLAB code and energy inventories to recalculate the CO_2_ emissions for China by adopting different emission factors.

### Energy-related reference approach emissions

Apart from the sectoral approach, the energy-related emissions of one region can also be estimated using the reference approach. *‘The Reference Approach is a top-down approach, using a country’s energy supply data to calculate the emissions of CO*_*2*_ *from combustion of mainly fossil fuels. The Reference Approach is a straightforward method that can be applied on the basis of relatively easily available energy supply statistics (Volume 2, Chapter 6, Page 5)’*^22^. The IPCC suggests ‘*to apply both a sectoral approach and the reference approach to estimate a country’s CO*_*2*_ *emissions from fuel combustion and to compare the results of these two independent estimates (Volume 2, Chapter6, Page 5)’*^22^. The reference emissions can be used to verify and support the sectoral emissions.

As the reference emissions were calculated from the fossil fuels’ production base, we only considered three primary fossil fuels (raw coal, crude oil, and natural gas). With the assumption of carbon balance, the carbon in the supply of the 3 primary fossil fuels should be equal to the carbon contained in the total consumption of the 17 fossil fuels^9^. We calculated the reference approach emissions as in equation (2):

(2)CEref−i=ADref−i×EFi

where *CE*_*ref−i*_ refers to the reference CO_2_ emissions from fossil fuel *i*, *EF*_*i*_ and *AD*_*ref−i*_ are the emission factors and apparent consumption of the corresponding fossil fuel, respectively. The emission factors for the 3 primary fossil fuels are the same as those used in the sectoral approach emissions calculation^10^. Values of *AD*_*ref−i*_ were calculated as in equation (3). For the same reason, we removed the non-energy use and loss parts from the fuel’s apparent consumption. The items in bracket were only used to calculate the apparent consumption of provinces and were skipped when calculating the national consumption.

(3)ADref−i=IndigenousProduction+Imports−Exports+(MovinginfromOtherProvinces−SendingOuttoOtherProvinces)±StockChange−Non–EnergyUse−Loss

All the items in equation (3) (at both the national and provincial scales) were collected from the most up to date energy balance tables published officially in the *China Energy Statistical Yearbooks*^25^.

### Process-related (cement) CO_2_ emissions

The process-related emissions refer to CO_2_ emitted as a result of physical-chemical reactions in the production process and not the energy combusted by the industry^28^. *‘The fossil fuels used in this transformation stage are considered the carbon emissions from fossil fuel combustion performed by the industrial sectors and are not considered as the industrial process emissions (page 240)’*^29^. In this study, we only investigated cement production, which accounts for approximately 75% of China’s total process-related CO_2_ emissions^7^. We calculated the cement-related CO_2_ emissions as in equation (4):

(4)CEt=ADt×EFt

where *CE*_*t*_ refers to the process-related CO_2_ emissions from cement production and *AD*_*t*_ is the activity data for cement-related emissions accounting, which refer to cement production. We collected data for the cement productions of China and its provinces from the official dataset of the National Bureau of Statistics^30^, which are consistent with the *China Statistical Yearbooks*^31^. The expression *EF*_*t*_ refers to the emission factor for cement production, which is 0.2906, also collected from Liu, *et al.*^10^. The cement-related CO_2_ emissions were allocated to the sector ‘Non-metal Mineral Products’ in the final emission inventories.

### Comparison of the sectoral- and reference-approach emission inventories

**Figure 2:**
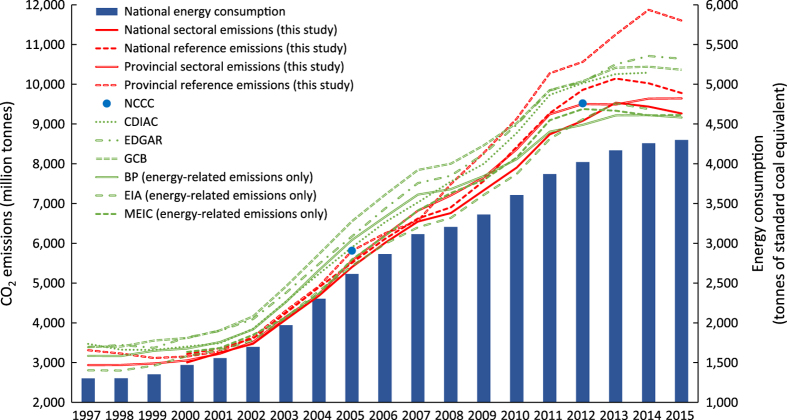
Comparisons of the two-approach emissions and other existing emission inventories. Data source: National energy consumption^12^; National Communication on Climate Change (NC) 2005^7^; NC2012^8^; Carbon Dioxide Information Analysis Centre (CDIAC)^42^; Emissions Database for Global Atmospheric Research (EDGAR)^43^; Global Carbon Budget (GCB)^44^; British Petroleum (BP)^45^; U.S. Energy Information Administration (EIA)^46^. Multi-resolution emission inventory for China (MEIC)^47–49^. Note that emissions by NC2005 include CO_2_ emissions from lime and glass production as well, emissions by MEIC, BP and EIA include the energy-related emissions only.

The difference between the sectoral- and reference-approach emission inventories laid in the way we calculated the fossil fuel consumptions when estimated the energy-related emissions. The process-related emissions from the two approaches were exactly the same. The sectoral emissions were calculated from the energy consumption aspect while the reference emissions were calculated via the energy production and trade data. The reference approach assumed that all the carbon elements from the primary energy sources (excluding the transport loss and non-energy usage part) were converted into CO_2_ emissions. IPCC suggest calculating the reference emissions for one country as a validation of the sectoral emissions. Therefore, we calculated both the sectoral and reference emission for China and its provinces in our datasets. The red lines in Fig. 2 compared the sectoral and reference emissions.

Our reference emissions were 1 to 7% higher than the sectoral emissions. The differences between the two approaches can be explained from three aspects. First, the energy loss during energy transformation process was not excluded from the reference energy consumption. Second, only transport loss and non-energy usage of primary energy sources were excluded from the total consumption in the reference approach. Those of secondary energy sources were not removed. Third, there was roughly 1.2% statistical difference between the energy production and consumption data in China’s energy balance table^12^.

As discussed in the energy-related reference approach emissions section above, the reference emissions were calculated with the data of primary fossil fuels only, while the emissions embodied in the secondary fossil fuels cannot be reflected. Due to the frequent energy trade among Chinese provinces, especially the secondary energy types, the provincial reference emissions cannot reflect the real CO_2_ emissions within one provincial boundary. Considering the data completeness and transparency, we provided the provincial reference emission inventories in our datasets as well for reference.

## Data Records

A total of 1,172 data records (emission and energy inventories) are contained in the datasets. Of these,

- 16 are national energy inventories (from 2000 to 2015) [Data Citation 1, File ‘China national energy inventory, 2000–2015’];
- 570 are provincial energy inventories (30 provinces, from 1997 to 2015) [Data Citation 1, File ‘China provincial energy inventory, 2000–2015’];
- 16 are national sectoral approach inventories (from 2000 to 2015) [Data Citation 1, File ‘China national CO_2_ emission inventory (sectoral approach), 2000–2015’];
- 16 are national reference approach inventories (from 2000 to 2015) [Data Citation 1, File ‘China national CO_2_ emission inventory (reference approach), 2000–2015’];
- 570 are provincial sectoral approach inventories (30 provinces, from 1997 to 2015) [Data Citation 1, File ‘China provincial CO_2_ emission inventory (sectoral approach), 1997–2015’];
- 570 are provincial reference approach inventories (30 provinces, from 1997 to 2015) [Data Citation 1, File ‘China provincial CO_2_ emission inventory (reference approach), 1997–2015’];

Our CO_2_ emission inventories were constructed in a uniform format. The sectoral approach emission inventories are matrices with 19 columns and 47 rows, as shown in Table 4 (available online only) (an example of the China CO_2_ emission inventory, 2015). The 19 columns are 17 fossil fuel-related emissions, cement-related emissions and total emissions. The 47 rows represent the 47 socioeconomic sectors. Each element of the matrices represents the CO_2_ emissions from fossil fuel combustion/cement production in the corresponding sector. The sectoral and reference approach inventories include emissions from every individual item (e.g., production and import) of the three primary energy sources and the cement process. As an example, Table 5 presents the sectoral and reference approach emission inventories for China from 2000 to 2015.

**Figure 3:**
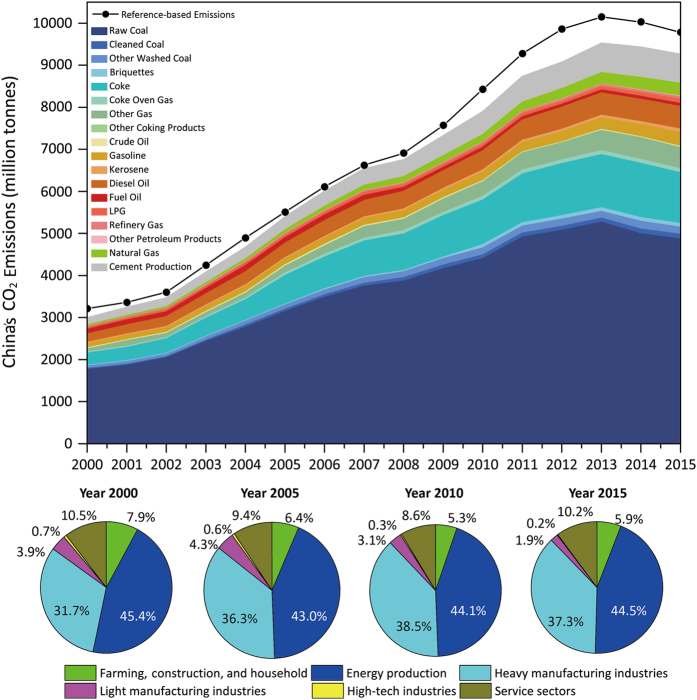
China’s CO_2_ emissions 2000 to 2015, in million tones. The stack area chart above represents CO_2_ emissions from 17 fossil fuels, and the black line represents the reference emissions. The chart shows that China’s total CO_2_ emissions peaked in 2013 (9,524.24 million tonnes, sectoral-based emissions; 10,145, reference-based emissions). Raw coal was the primary source of CO_2_ emissions, accounting for 52.58% of the total emissions in 2015. The four pie charts below illustrate the sectoral structure in CO_2_ emissions in 2000, 2005, 2010, and 2015. The energy production and heavy manufacturing industries were the primary contributors.

Figure 3 represents China’s CO_2_ emissions by fossil fuel types since 2000 and the sector structure of 2000, 2005, 2010, and 2015. Table 6 and 7 show the sectoral and reference approach emissions of China's 30 provinces (excluding the Tibet, Hong Kong, Macro and Taiwan due to data lacking).

## Technical Validation

### Uncertainty analysis

Uncertainty analyses are an important tool for improving emission inventories with uncertainty, which are an essential element of a greenhouse gas emissions inventory. Considering the small amounts and low uncertainties of the process-related emissions in cement production^10,32^, we only calculated the uncertainties from energy-related emissions in this study. The uncertainties of inventory are caused by many reasons, as the energy-related CO_2_ emissions were calculated as fossil fuel consumption (activity data) multiplied by the emission factors, the uncertainties should be ‘*derived for the component parts such as emission factors, activity data and other estimation parameters (Volume 1, Chapter 3, Page 6)*’^22^. We quantified both the uncertainties of emission factors and fossil fuel consumption data for our datasets.

As introduced above in the Methods section, this study adopted the emission factors from Liu, *et al.*^10^. However, the emission factors of China’s fossil fuel combustions may have large variations as discussed in subsequent studies (such as Olivier, *et al.*^33^; Le Quéré, *et al.*^34^; Korsbakken, *et al.*^35^; Jackson, *et al.*^36^). To quantitatively characterize the range of emission factor, we summarised the emission factors (*NCV*_*i*_, *CC*_*i*_, and *O*_*i*_) from seven other sources: IPCC, National Bureau of Statistics (NBS), NDRC, Initial National Communication on Climate Change (NC1994), Second National Communication on Climate Change (NC2005), Multi-resolution emission inventory for China (MEIC), UN-China, and UN-average (shown in Table 8 (available online only)). It is found that the fuels’ net caloric values varied a larger range than those of carbon content and oxygenation efficiency. Taking raw coal as an example, the Coefficient of Variation (CV, the standard deviation divided by the mean) of raw coal’s net caloric value is 15%, while the CVs of carbon content and oxygenation efficiency are 2 and 4% respectively. The CV of raw coal’s comprehensive emission factor (*NCV*_*i*_×*CC*_*i*_×*O*_*i*_) is 18%. The emission factor of coal-related fuels varied in a wider range than those of oil-related fuels and the natural gas. The average CV of coal-related fuels is 18%, while that for the oil-related fuels and natural gas is 4 and 5% respectively. Among the emission factors from eight sources, the IPCC and UN-average have the highest values, while Liu *et al.*’s study (used in this study), MEIC and NC1994 have the lowest values.

Due to the poor quality of China’s fossil fuel data, the fossil fuel consumption data also have large uncertainties. According to the previous literature, the fossil fuel consumed in electricity generation sector had a CV of 5%^37,38^, while the fossil fuel consumed in other industry and construction sector had a CV of 10%^22,39^. The CV of fossil fuel consumed in the transportation sector was 16%^40^, while residential and primary industry fossil fuel usage even had higher CVs of 20%^22^ and 30%^41^ respectively. The uncertainties in China’s fossil fuel data has been addressed and discussed by Guan, *et al.*^11^ previously. Possible reasons include the opaqueness in China’s statistical systems, especially on the ‘*statistical approach on data collection, reporting and validation (Page 673)*’^11^; and the dependence of China’s statistics departments with other government departments. As a result, China’s national fossil fuel consumption is smaller than the provincial aggregated data. Despite that China enlarged its 2000–2013 national energy data in 2014, there was still roughly 5% gap between the latest national and provincial aggregated energy data.

We employed the Monte Carlo simulations to propagate the uncertainties induced by both fossil fuel consumption and emission factors to provide the uncertainty estimates for entire emission inventories^22^. According to the Monte Carlo technique, we first assumed normal distributions (probability density functions) for both activity data (fossil fuel consumption) and emission factors with CVs discussed above^10,32^. Random sampling on both the activity data and emission factors were then conducted for 100,000 times and generated 100,000 estimations on the CO_2_ emissions. The uncertainty range, therefore, was 97.5% confidential intervals of the estimations. The above simulation was conducted in MATLAB R2014a.

**Figure 4:**
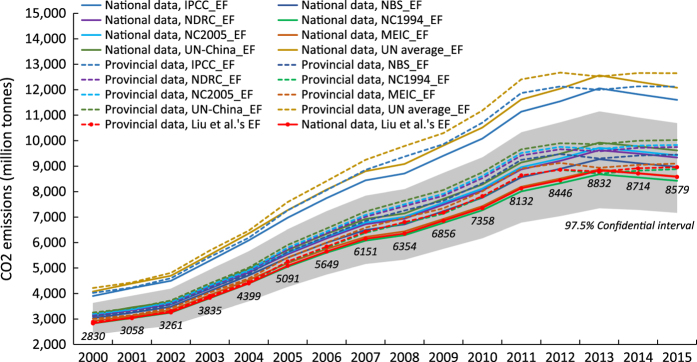
Uncertainties of the emissions. The grey area in the figure shows the 97.5% confidential interval of China’s CO_2_ emission estimations. The lines present China’s CO_2_ emission calculated based on the national/provincial aggregated energy data and different emission factors. The figure shows that emissions calculated based on the NBS, NDRC, NC1994, NC2005, MEIC, UN-China’s emission factors fall in the 97.5% confidential interval.

We found that the uncertainties of the entire CO_2_ emissions inventories were roughly (−15%, 25%) at a 97.5% confidential level. Table 9 (available online only) and Fig. 4 show the uncertainties in the national emission inventories from 2000 to 2015. The above ranges, e.g., (−15%, 25%), reflected the uncertainties from both emission factor and activity data. In particular, concerning the continuous debate on the emission factor of fossil fuel combustion in China^33–36^, we incorporated 8 emission factors from independent sources to represent the uncertainty of emission factors. In order to separate the uncertainty induced by emission factor and activity data, we then conducted the Monte Carlo simulations by assuming the CV of one of them was 0. The results showed that uncertainties from the emission factors in 2015 were (−15.8%, 23.7%), while the uncertainties from the activity data were (−1.4%, 9.2%). This implied the emission factors of fossil fuels induced higher uncertainty to the final estimation.

In Fig. 4, the grey area in the figure indicates the 97.5% confidential interval of China’s CO_2_ emission estimations of this study. The solid lines present China’s CO_2_ emission calculated based on the national energy data and 8 different emission factors, while the dash lines present the emissions based on the provincial aggregated energy data. The figure shows that emissions calculated based on emission factors from Liu *et al.*’s nature, NBS, NDRC, NC1994, NC2005, MEIC, and UN-China’s fall in the 97.5% confidential interval. Emissions calculated based on the IPCC and UN-average emissions factors are 10% larger than the upper bound of the 97.5% confidential interval due to their high emission factor value, while the emissions calculated based on the emission factors from Liu *et al.*’s nature, NC1994, and MEIC have relatively low values. In addition, the emissions calculated based the provincial aggregated energy data are about 5% higher than that based on national data due to the difference in the national and provincial data.

In addition to the uncertainties of emission factors and fossil fuel data considered in the Monte Carlo techniques above, there were some other uncertainties that should be taken into consideration when using the datasets, such as ‘lack of completeness’, ‘lack of data’, ‘measurement error’. These uncertainties were very small and difficult to quantify; however, they were also essential parts of the inventories’ uncertainties. 1) Lack of completeness: We only considered the energy-related emissions and cement-related emissions in our datasets. Emissions from other sources were not taken into account, such as ‘agriculture’, ‘land-use change and forestry’, ‘waste’, and other industrial processes. 2) Lake of data: As discussed above, the sectoral fossil fuel consumption of 8 provinces were lacking. We used the sectoral fossil fuel consumption structure in 2008 to estimate that of the intervening years. Such a replacement had no much effect on the total emissions, but increased the uncertainties in provincial sectoral emissions. Also, the emission factors for secondary fossil fuels were estimated based on the primary fossil fuel emission factors’ ratio. 3) Measurement error: the *‘measurement error is random or systematic, results from errors in measuring, recording and transmitting information; inexact values of constants and other parameters obtained from external sources (Volume 1, Chapter 3, Page 11)’*^22^. The measurement errors might be generated in the energy statistics and emission factors’ calculation.

### Comparison with existing emission estimates

We compared our emissions with estimates from other research institutes, shown in Fig. 2. We found that our national sectoral emissions were the lowest among the estimates. The Global Carbon Budget (GCB) had the highest value until EDGAR passed it since 2012. Our national sectoral emissions were 9 to 18% lower than the highest value. This was mainly because that we used the updated emission factors, which were lower than the IPCC default value. Our results were 1–3% higher than BP and MEIC’s since 2013. Even considering the emissions from BP and MEIC not including the cement-related emissions, they had closer results with our datasets compared with other emission estimations. Our estimates were highly consistent with the newly published official emission inventory. The Chinese government published the ‘First Biennial Update Report on Climate Change^8^’ by the end of 2016. In the report, the energy-related CO_2_ emissions in 2012 were 8,688 million tonnes (the blue points in Fig. 2), only 2.79% higher than our estimates (national sectoral emissions, 8,446 million tonnes). This tiny difference falls into the uncertainty range of the both inventories.

From the aspect of format, the existing emission estimates only present the total energy-related emissions of the whole country, or emissions from three fossil fuel categories at most (solid, liquid, and gas). Our datasets provided the energy-related CO_2_ emissions from 47 socioeconomic sectors and 17 fossil fuels to give detailed demonstrations of China’s emission statue as well as its provinces. Thus, our datasets can be a more detailed supplement to the existing emission estimates and the official emission inventories.

### Limitations

Our datasets have the following limitations: 1) We used the national average emission factors of fossil fuels and cement production when calculating the provincial CO_2_ emissions in the current version. The emission factors should be different in different regions considering the discrepancy in energy quality and cement production technology. In the future research, we will specify the emission factor of each province to achieve more accurate emission inventories for provinces; 2) In the current version, we used the sectoral fossil fuel consumption structure in 2008 to estimate that of the intervening years for 8 certain provinces. In the future, we will investigate the 8 provinces for more accurate data. 3) We only considered emissions from cement production in the current process-related emissions accounts. The latest official emission inventory in 2012 include other 9 processes such as glass, lime, steel production. In the future research, we will extend the scope of our datasets to include more industrial processes.

## Additional information

**How to cite this article:** Shan, Y. *et al.* China CO_2_ emission accounts 1997–2015. *Sci. Data* 5:170201 doi:10.1038/sdata.2017.201 (2018).

**Publisher’s note:** Springer Nature remains neutral with regard to jurisdictional claims in published maps and institutional affiliations.

## Supplementary Material



Supplementary Information

## Figures and Tables

**Table 1:** Fossil fuels and emission factors (*NCV*_*i*_, *CC*_*i*_).

**No. (i)Unit**	**Fuels in China’s Energy Statistics**	**Fuels in this study**	***NCV***_***i***_	***CC***_***i***_
			***PJ*****/10**^**4**^ ***tonnes*****, 10**^**8**^***m***^**3**^	***tonne*****C/*****TJ***
1	Raw coal	Raw coal	0.21	26.32
2	Cleaned coal	Cleaned coal	0.26	26.32
3	Other washed coal	Other washed coal	0.15	26.32
4	Briquettes	Briquette	0.18	26.32
	Gangue			
5	Coke	Coke	0.28	31.38
6	Coke oven gas	Coke over gas	1.61	21.49
7	Blast furnace gas	Other gas	0.83	21.49
	Converter gas			
	Other gas			
8	Other coking products	Other coking products	0.28	27.45
9	Crude Oil	Crude oil	0.43	20.08
10	Gasoline	Gasoline	0.44	18.90
11	Kerosene	Kerosene	0.44	19.60
12	Diesel oil	Diesel oil	0.43	20.20
13	Fuel oil	Fuel oil	0.43	21.10
14	Naphtha	Other petroleum products	0.51	17.2
	Lubricants			
	Paraffin			
	White spirit			
	Bitumen asphalt			
	Petroleum coke			
	Other petroleum products			
15	Liquefied petroleum gas (LPG)	LPG	0.47	20.00
16	Refinery gas	Refinery gas	0.43	20.20
17	Nature gas	Nature gas	3.89	15.32

**Table 2:** Economic sectors.

**No. (j)**	**Socioeconomic sectors**	**Category**	
1	Farming, Forestry, Animal Husbandry, Fishery and Water Conservancy	The primary industry	
2	Coal Mining and Dressing	Energy production	Manufacturing industries
3	Petroleum and Natural Gas Extraction	Energy production	
4	Ferrous Metals Mining and Dressing	Energy production	
5	Nonferrous Metals Mining and Dressing	Energy production	
6	Non-metal Minerals Mining and Dressing	Energy production	
7	Other Minerals Mining and Dressing	Energy production	
8	Logging and Transport of Wood and Bamboo	Light manufacturing	
9	Food Processing	Light manufacturing	
10	Food Production	Light manufacturing	
11	Beverage Production	Light manufacturing	
12	Tobacco Processing	Light manufacturing	
13	Textile Industry	Light manufacturing	
14	Garments and Other Fibre Products	Light manufacturing	
15	Leather, Furs, Down and Related Products	Light manufacturing	
16	Timber Processing, Bamboo, Cane, Palm Fibre & Straw Products	Light manufacturing	
17	Furniture Manufacturing	Light manufacturing	
18	Papermaking and Paper Products	Light manufacturing	
19	Printing and Record Medium Reproduction	Light manufacturing	
20	Cultural, Educational and Sports Articles	Light manufacturing	
21	Petroleum Processing and Coking	Energy production	
22	Raw Chemical Materials and Chemical Products	Heavy manufacturing	
23	Medical and Pharmaceutical Products	Light manufacturing	
24	Chemical Fibre	Heavy manufacturing	
25	Rubber Products	Heavy manufacturing	
26	Plastic Products	Heavy manufacturing	
27	Non-metal Mineral Products	Heavy manufacturing	
28	Smelting and Pressing of Ferrous Metals	Heavy manufacturing	
29	Smelting and Pressing of Nonferrous Metals	Heavy manufacturing	
30	Metal Products	Heavy manufacturing	
31	Ordinary Machinery	Heavy manufacturing	
32	Equipment for Special Purposes	Heavy manufacturing	
33	Transportation Equipment manufacturing	Heavy manufacturing	
34	Electric Equipment and Machinery	High-tech industry	
35	Electronic and Telecommunications Equipment	High-tech industry	
36	Instruments, Meters, Cultural and Office Machinery	High-tech industry	
37	Other Manufacturing Industry	High-tech industry	
38	Scrap and waste	High-tech industry	
39	Production and Supply of Electric Power, Steam and Hot Water	Energy production	
40	Production and Supply of Gas	Energy production	
41	Production and Supply of Tap Water	Heavy manufacturing	
42	Construction	Construction	
43	Transportation, Storage, Post and Telecommunication Services	Services sectors	
44	Wholesale, Retail Trade and Catering Services		
45	Other Service Sectors		
46	Urban Resident Energy Usage	Household	
47	Rural Resident Energy Usage		

**Table 3:** Oxygenation efficiency of fossil fuels combusted in sectors (*O*_*ij*_)

**Sectors**	**Raw Coal**	**Cleaned Coal**	**Other Washed Coal**	**Briquettes**	**Coke**	**Coke Oven Gas**	**Other Gas**	**Other Coking Products**	**Crude Oil**	**Gasoline**	**Kerosene**	**Diesel Oil**	**Fuel Oil**	**LPG**	**Refinery Gas**	**Other Petroleum Products**	**Natural Gas**
Farming, Forestry, Animal Husbandry, Fishery and Water Conservancy	83%	83%	83%	83%	89%	91%	91%	89%	96%	96%	96%	96%	96%	97%	97%	96%	98%
Coal Mining and Dressing	82%	82%	82%	82%	89%	91%	91%	89%	96%	96%	96%	96%	96%	97%	97%	96%	98%
Petroleum and Natural Gas Extraction	82%	82%	82%	82%	89%	91%	91%	89%	96%	96%	96%	96%	96%	97%	97%	96%	98%
Ferrous Metals Mining and Dressing	82%	82%	82%	82%	89%	91%	91%	89%	96%	96%	96%	96%	96%	97%	97%	96%	98%
Nonferrous Metals Mining and Dressing	82%	82%	82%	82%	89%	91%	91%	89%	96%	96%	96%	96%	96%	97%	97%	96%	98%
Non-metal Minerals Mining and Dressing	82%	82%	82%	82%	89%	91%	91%	89%	96%	96%	96%	96%	96%	97%	97%	96%	98%
Other Minerals Mining and Dressing	82%	82%	82%	82%	89%	91%	91%	89%	96%	96%	96%	96%	96%	97%	97%	96%	98%
Logging and Transport of Wood and Bamboo	82%	82%	82%	82%	89%	91%	91%	89%	96%	96%	96%	96%	96%	97%	97%	96%	98%
Food Processing	80%	80%	80%	80%	89%	91%	91%	89%	96%	96%	96%	96%	96%	97%	97%	96%	98%
Food Production	80%	80%	80%	80%	89%	91%	91%	89%	96%	96%	96%	96%	96%	97%	97%	96%	98%
Beverage Production	80%	80%	80%	80%	89%	91%	91%	89%	96%	96%	96%	96%	96%	97%	97%	96%	98%
Tobacco Processing	80%	80%	80%	80%	89%	91%	91%	89%	96%	96%	96%	96%	96%	97%	97%	96%	98%
Textile Industry	82%	82%	82%	82%	89%	91%	91%	89%	96%	96%	96%	96%	96%	97%	97%	96%	98%
Garments and Other Fibre Products	82%	82%	82%	82%	89%	91%	91%	89%	96%	96%	96%	96%	96%	97%	97%	96%	98%
Leather, Furs, Down and Related Products	82%	82%	82%	82%	89%	91%	91%	89%	96%	96%	96%	96%	96%	97%	97%	96%	98%
Timber Processing, Bamboo, Cane, Palm Fibre & Straw Products	80%	80%	80%	80%	89%	91%	91%	89%	96%	96%	96%	96%	96%	97%	97%	96%	98%
Furniture Manufacturing	80%	80%	80%	80%	89%	91%	91%	89%	96%	96%	96%	96%	96%	97%	97%	96%	98%
Papermaking and Paper Products	80%	80%	80%	80%	89%	91%	91%	89%	96%	96%	96%	96%	96%	97%	97%	96%	98%
Printing and Record Medium Reproduction	80%	80%	80%	80%	89%	91%	91%	89%	96%	96%	96%	96%	96%	97%	97%	96%	98%
Cultural, Educational and Sports Articles	80%	80%	80%	80%	89%	91%	91%	89%	96%	96%	96%	96%	96%	97%	97%	96%	98%
Petroleum Processing and Coking	83%	83%	83%	83%	89%	91%	91%	89%	96%	96%	96%	96%	96%	97%	97%	96%	98%
Raw Chemical Materials and Chemical Products	85%	85%	85%	85%	89%	91%	91%	89%	96%	96%	96%	96%	96%	97%	97%	96%	98%
Medical and Pharmaceutical Products	85%	85%	85%	85%	89%	91%	91%	89%	96%	96%	96%	96%	96%	97%	97%	96%	98%
Chemical Fibre	85%	85%	85%	85%	89%	91%	91%	89%	96%	96%	96%	96%	96%	97%	97%	96%	98%
Rubber Products	85%	85%	85%	85%	89%	91%	91%	89%	96%	96%	96%	96%	96%	97%	97%	96%	98%
Plastic Products	85%	85%	85%	85%	89%	91%	91%	89%	96%	96%	96%	96%	96%	97%	97%	96%	98%
Non-metal Mineral Products	90%	90%	90%	90%	89%	91%	91%	89%	96%	96%	96%	96%	96%	97%	97%	96%	98%
Smelting and Pressing of Ferrous Metals	84%	84%	84%	84%	89%	91%	91%	89%	96%	96%	96%	96%	96%	97%	97%	96%	98%
Smelting and Pressing of Nonferrous Metals	84%	84%	84%	84%	89%	91%	91%	89%	96%	96%	96%	96%	96%	97%	97%	96%	98%
Metal Products	82%	82%	82%	82%	89%	91%	91%	89%	96%	96%	96%	96%	96%	97%	97%	96%	98%
Ordinary Machinery	82%	82%	82%	82%	89%	91%	91%	89%	96%	96%	96%	96%	96%	97%	97%	96%	98%
Equipment for Special Purposes	82%	82%	82%	82%	89%	91%	91%	89%	96%	96%	96%	96%	96%	97%	97%	96%	98%
Transportation Equipment	82%	82%	82%	82%	89%	91%	91%	89%	96%	96%	96%	96%	96%	97%	97%	96%	98%
Electric Equipment and Machinery	82%	82%	82%	82%	89%	91%	91%	89%	96%	96%	96%	96%	96%	97%	97%	96%	98%
Electronic and Telecommunications Equipment	82%	82%	82%	82%	89%	91%	91%	89%	96%	96%	96%	96%	96%	97%	97%	96%	98%
Instruments, Meters, Cultural and Office Machinery	82%	82%	82%	82%	89%	91%	91%	89%	96%	96%	96%	96%	96%	97%	97%	96%	98%
Other Manufacturing Industry	82%	82%	82%	82%	89%	91%	91%	89%	96%	96%	96%	96%	96%	97%	97%	96%	98%
Scrap and Waste	87%	87%	87%	87%	89%	91%	91%	89%	96%	96%	96%	96%	96%	97%	97%	96%	98%
Production and Supply of Electric Power, Steam and Hot Water	87%	87%	87%	87%	89%	91%	91%	89%	96%	96%	96%	96%	96%	97%	97%	96%	98%
Production and Supply of Gas	82%	82%	82%	82%	89%	91%	91%	89%	96%	96%	96%	96%	96%	97%	97%	96%	98%
Production and Supply of Tap Water	82%	82%	82%	82%	89%	91%	91%	89%	96%	96%	96%	96%	96%	97%	97%	96%	98%
Construction	83%	83%	83%	83%	89%	91%	91%	89%	96%	96%	96%	96%	96%	97%	97%	96%	98%
Transportation, Storage, Post and Telecommunication Services	74%	74%	74%	74%	89%	91%	91%	89%	96%	96%	96%	96%	96%	97%	97%	96%	98%
Wholesale, Retail Trade and Catering Services	74%	74%	74%	74%	89%	91%	91%	89%	96%	96%	96%	96%	96%	97%	97%	96%	98%
Others	74%	74%	74%	74%	89%	91%	91%	89%	96%	96%	96%	96%	96%	97%	97%	96%	98%
Urban Household Energy Use	74%	74%	74%	74%	89%	91%	91%	89%	96%	96%	96%	96%	96%	97%	97%	96%	98%
Rural Household Energy Use	74%	74%	74%	74%	89%	91%	91%	89%	96%	96%	96%	96%	96%	97%	97%	96%	98%

**Table 4:** Sectoral approach CO_2_ emission inventory of China, 2015 (in million tonnes)

**Sectors**	**Total Emissions**	**Raw Coal**	**Cleaned Coal**	**Other Washed Coal**	**Briquettes**	**Coke**	**Coke Oven Gas**	**Other Gas**	**Other Coking Products**	**Crude Oil**	**Gasoline**	**Kerosene**	**Diesel Oil**	**Fuel Oil**	**LPG**	**Refinery Gas**	**Other Petroleum Products**	**Natural Gas**	**Process**
Total Consumption	9265.06	4872.02	111.65	171.96	76.90	1217.12	92.75	493.47	17.22	20.01	332.44	80.79	534.66	69.34	104.82	57.60	9.97	316.77	685.58
Farming, Forestry, Animal Husbandry, Fishery and Water Conservancy	97.84	42.43	0.00	0.53	0.00	1.42	0.00	0.00	0.00	0.00	6.77	0.03	46.19	0.03	0.22	0.00	0.00	0.21	0.00
Coal Mining and Dressing	80.21	58.45	2.28	8.35	0.88	1.81	0.49	0.86	0.05	0.00	0.31	0.05	5.10	0.01	0.00	0.00	0.00	1.54	0.00
Petroleum and Natural Gas Extraction	45.51	2.25	0.00	0.00	0.03	0.00	0.00	0.00	0.00	13.23	0.33	0.00	1.47	0.71	0.01	0.82	0.00	26.66	0.00
Ferrous Metals Mining and Dressing	14.81	4.40	1.35	0.02	0.07	4.53	0.28	1.47	0.00	0.00	0.11	0.00	2.56	0.00	0.00	0.00	0.00	0.00	0.00
Nonferrous Metals Mining and Dressing	4.28	2.16	0.33	0.01	0.03	0.25	0.06	0.00	0.00	0.00	0.21	0.01	0.95	0.05	0.00	0.00	0.00	0.23	0.00
Non-metal Minerals Mining and Dressing	11.97	6.77	0.32	2.20	0.10	0.20	0.04	0.00	0.00	0.00	0.10	0.01	2.19	0.01	0.00	0.00	0.00	0.02	0.00
Other Minerals Mining and Dressing	4.03	0.53	0.00	0.00	0.01	0.00	0.00	0.00	0.00	0.01	0.12	0.00	2.90	0.01	0.00	0.00	0.00	0.44	0.00
Logging and Transport of Wood and Bamboo	0.00	0.00	0.00	0.00	0.00	0.00	0.00	0.00	0.00	0.00	0.00	0.00	0.00	0.00	0.00	0.00	0.00	0.00	0.00
Food Processing	42.79	32.80	0.42	0.38	0.50	4.06	0.03	1.84	0.00	0.00	0.83	0.02	1.48	0.06	0.04	0.00	0.00	0.33	0.00
Food Production	20.71	17.41	0.14	0.21	0.26	0.09	0.02	0.00	0.00	0.00	0.30	0.00	0.49	0.11	0.04	0.00	0.00	1.63	0.00
Beverage Production	20.00	17.74	0.10	0.14	0.27	0.03	0.02	0.00	0.00	0.00	0.19	0.00	0.35	0.02	0.02	0.00	0.00	1.12	0.00
Tobacco Processing	0.95	0.58	0.06	0.01	0.01	0.00	0.00	0.00	0.00	0.00	0.02	0.00	0.06	0.01	0.00	0.00	0.00	0.20	0.00
Textile Industry	27.73	24.60	0.10	0.12	0.37	0.06	0.30	0.00	0.00	0.00	0.41	0.00	0.45	0.23	0.08	0.00	0.00	1.02	0.00
Garments and Other Fibre Products	4.74	3.64	0.01	0.02	0.05	0.05	0.00	0.00	0.00	0.00	0.35	0.00	0.43	0.02	0.03	0.00	0.00	0.14	0.00
Leather, Furs, Down and Related Products	2.97	2.47	0.02	0.01	0.04	0.01	0.00	0.00	0.00	0.00	0.20	0.00	0.17	0.03	0.01	0.00	0.00	0.01	0.00
Timber Processing, Bamboo, Cane, Palm Fibre & Straw Products	8.68	7.75	0.01	0.02	0.12	0.04	0.01	0.00	0.00	0.00	0.21	0.02	0.37	0.01	0.00	0.00	0.00	0.14	0.00
Furniture Manufacturing	1.47	0.79	0.00	0.01	0.01	0.05	0.00	0.00	0.00	0.00	0.15	0.00	0.22	0.01	0.02	0.00	0.00	0.20	0.00
Papermaking and Paper Products	27.54	25.29	0.32	0.27	0.38	0.02	0.00	0.00	0.00	0.00	0.18	0.00	0.54	0.17	0.02	0.00	0.00	0.34	0.00
Printing and Record Medium Reproduction	2.15	1.27	0.00	0.01	0.02	0.01	0.00	0.00	0.01	0.00	0.19	0.00	0.20	0.01	0.02	0.00	0.00	0.40	0.00
Cultural, Educational and Sports Articles	2.80	1.76	0.07	0.02	0.03	0.10	0.00	0.00	0.00	0.00	0.24	0.00	0.24	0.03	0.03	0.00	0.00	0.29	0.00
Petroleum Processing and Coking	158.71	34.31	13.95	12.75	0.52	1.86	12.04	5.82	3.34	2.15	0.09	0.00	0.48	3.53	10.86	49.15	0.00	7.88	0.00
Raw Chemical Materials and Chemical Products	307.31	199.71	10.62	0.00	4.91	52.72	4.41	1.29	4.30	4.02	0.90	0.05	1.36	0.83	0.35	1.45	0.00	20.37	0.00
Medical and Pharmaceutical Products	13.34	11.78	0.08	0.00	0.29	0.01	0.00	0.00	0.00	0.00	0.27	0.00	0.23	0.01	0.00	0.00	0.00	0.65	0.00
Chemical Fibre	6.39	5.98	0.10	0.00	0.15	0.00	0.00	0.00	0.00	0.00	0.02	0.00	0.05	0.02	0.00	0.00	0.00	0.07	0.00
Rubber Products	4.72	3.67	0.09	0.00	0.09	0.03	0.01	0.00	0.00	0.00	0.26	0.00	0.28	0.02	0.00	0.00	0.00	0.26	0.00
Plastic Products	4.72	3.67	0.09	0.00	0.09	0.03	0.01	0.00	0.00	0.00	0.26	0.00	0.28	0.02	0.00	0.00	0.00	0.26	0.00
Non-metal Mineral Products	1273.96	416.16	14.02	86.18	6.28	26.01	4.18	2.96	1.85	0.01	0.88	0.07	9.07	6.57	2.30	0.01	0.00	11.82	685.58
Smelting and Pressing of Ferrous Metals	1690.19	164.26	58.50	4.28	2.48	1065.99	39.82	336.77	7.35	0.00	0.33	0.01	2.09	0.12	0.55	0.05	0.00	7.59	0.00
Smelting and Pressing of Nonferrous Metals	65.32	31.42	4.16	1.03	0.47	16.24	1.13	3.52	0.25	0.00	0.20	0.02	1.37	1.41	0.15	0.00	0.00	3.94	0.00
Metal Products	14.38	7.01	0.23	0.04	0.11	2.88	0.26	0.29	0.00	0.00	0.65	0.03	0.93	0.21	0.18	0.00	0.00	1.57	0.00
Ordinary Machinery	28.84	4.54	0.10	0.02	0.07	19.68	0.03	0.25	0.00	0.00	0.91	0.08	1.12	0.04	0.09	0.00	0.00	1.90	0.00
Equipment for Special Purposes	10.33	3.35	1.29	0.02	0.05	1.95	0.12	0.15	0.01	0.00	0.76	0.03	1.45	0.04	0.10	0.00	0.00	0.99	0.00
Transportation Equipment	18.01	5.96	0.06	0.27	0.09	3.80	0.06	0.07	0.00	0.00	1.24	0.08	1.79	0.15	0.18	0.00	0.00	4.26	0.00
Electric Equipment and Machinery	6.83	3.52	0.34	0.03	0.05	0.35	0.00	0.00	0.01	0.00	0.77	0.02	0.76	0.06	0.24	0.00	0.00	0.68	0.00
Electronic and Telecommunications Equipment	4.32	1.36	0.02	0.01	0.02	0.41	0.00	0.00	0.00	0.00	0.43	0.01	0.41	0.07	0.06	0.00	0.00	1.53	0.00
Instruments, Meters, Cultural and Office Machinery	0.88	0.32	0.00	0.01	0.00	0.08	0.00	0.00	0.00	0.00	0.17	0.01	0.13	0.01	0.01	0.00	0.00	0.13	0.00
Other Manufacturing Industry	1.58	1.03	0.00	0.02	0.02	0.01	0.00	0.00	0.00	0.00	0.08	0.04	0.18	0.02	0.13	0.00	0.00	0.06	0.00
Scrap and Waste	2.40	1.02	0.00	0.06	0.02	0.62	0.05	0.21	0.05	0.00	0.02	0.00	0.13	0.02	0.01	0.00	0.00	0.18	0.00
Production and Supply of Electric Power, Steam and Hot Water	3836.69	3468.47	2.12	43.20	50.71	9.22	26.59	133.97	0.00	0.58	0.76	0.00	2.05	6.26	0.10	6.11	9.97	76.57	0.00
Production and Supply of Gas	1.43	0.14	0.00	0.01	0.00	0.00	0.27	0.01	0.00	0.00	0.10	0.00	0.08	0.01	0.01	0.00	0.00	0.80	0.00
Production and Supply of Tap Water	0.45	0.21	0.00	0.00	0.00	0.00	0.00	0.00	0.00	0.00	0.12	0.00	0.07	0.00	0.00	0.00	0.00	0.05	0.00
Construction	46.75	14.00	0.17	0.23	0.00	0.19	0.00	0.00	0.00	0.00	11.96	0.38	17.19	1.70	0.47	0.00	0.00	0.47	0.00
Transportation, Storage, Post and Telecommunication Services	673.86	6.74	0.17	0.23	0.00	0.09	0.00	0.00	0.00	0.00	155.31	76.03	345.36	45.61	3.13	0.00	0.00	41.19	0.00
Wholesale, Retail Trade and Catering Services	87.60	55.11	0.00	0.67	0.43	1.15	0.10	0.37	0.00	0.00	7.12	0.35	7.97	0.60	2.63	0.00	0.00	11.08	0.00
Others	180.96	59.16	0.00	1.10	0.16	0.15	0.14	0.00	0.00	0.00	61.71	2.53	42.82	0.51	2.86	0.00	0.00	9.82	0.00
Urban Household Energy Use	233.26	14.49	0.00	2.19	2.09	0.26	2.27	3.60	0.00	0.00	52.80	0.11	17.01	0.00	60.99	0.00	0.00	77.44	0.00
Rural Household Energy Use	170.70	101.52	0.00	7.27	4.62	0.63	0.00	0.01	0.00	0.00	23.09	0.77	13.64	0.00	18.83	0.00	0.00	0.31	0.00

**Table 5:** Sectoral and reference approach CO_2_ emission inventory of China, 2000–2015 (in million tonnes)

**Items**		**2000**	**2001**	**2002**	**2003**	**2004**	**2005**	**2006**	**2007**	**2008**	**2009**	**2010**	**2011**	**2012**	**2013**	**2014**	**2015**
Raw Coal	Indigenous production	2532.6	2692.4	2836.7	3357.3	3883.7	4327.4	4701.8	5049.7	5312.3	5700.1	6272.9	6887.7	7218.3	7271.7	7088.0	6854.9
	Import	4.1	5.0	21.0	20.7	34.7	48.8	71.2	96.1	81.3	245.6	340.9	414.0	537.1	609.0	542.4	380.0
	Export(−)	−100.8	−164.9	−153.5	−172.0	−158.6	−131.2	−115.8	−97.3	−83.4	−41.0	−34.8	−26.6	−16.7	−13.5	−10.3	−9.5
	Stock decrease	−23.8	3.9	14.9	49.8	−0.3	65.2	127.9	129.6	76.1	−19.0	−63.7	−53.0	−52.6	−48.1	−60.9	33.6
	Loss	0.0	0.0	0.0	0.0	0.0	0.0	0.0	0.0	0.0	0.0	0.0	0.0	0.0	0.0	0.0	0.0
	Non-energy use	58.4	61.5	64.0	76.0	94.3	107.4	115.4	114.6	119.6	124.3	129.6	127.8	139.7	151.1	141.3	152.9
	Raw coal total	2353.7	2474.8	2655.1	3179.7	3665.2	4202.8	4669.6	5063.5	5266.6	5761.5	6385.7	7094.3	7546.4	7668.1	7418.0	7106.2
Crude Oil	Indigenous production	500.8	503.8	513.1	521.1	540.4	557.2	567.7	572.6	585.2	582.2	623.8	623.4	637.5	645.0	649.7	659.3
	Import	215.9	185.2	213.3	279.7	377.1	389.7	446.1	501.3	549.7	625.8	730.3	779.8	832.8	865.7	947.5	1030.8
	Export(−)	−31.7	−23.2	−23.6	−25.0	−16.9	−24.8	−19.5	−11.9	−13.0	−15.6	−9.3	−7.7	−7.5	−5.0	−1.8	−8.8
	Stock decrease	−28.1	−4.0	−3.2	−1.9	−9.2	2.4	−3.4	−16.1	−31.0	−20.8	−27.3	−44.7	−28.4	−10.3	−11.5	−19.2
	Loss	5.9	5.8	5.8	4.9	4.6	4.7	6.1	6.1	6.2	5.7	5.9	5.4	5.5	6.5	3.3	2.7
	Non-energy use	2.5	2.6	2.7	3.2	3.5	3.5	6.0	5.3	4.4	5.0	4.8	2.5	1.1	2.7	11.2	4.2
	Crude oil total	648.7	653.4	691.1	765.7	883.4	916.3	978.8	1034.5	1080.2	1160.9	1306.8	1342.9	1427.9	1486.2	1569.3	1655.3
Natural Gas	Indigenous production	58.8	65.6	70.7	75.7	89.7	106.7	126.7	149.8	173.7	184.5	207.2	227.9	239.3	261.5	281.6	291.2
	Import	0.0	0.0	0.0	0.0	0.0	0.0	2.1	8.7	10.0	16.5	7.7	31.0	47.2	59.9	68.7	73.7
	Export(−)	−6.8	−6.6	−6.9	−4.1	−5.3	−6.4	−6.3	−5.6	−7.0	−6.9	−8.7	−6.9	−6.3	−5.9	−5.6	−7.0
	Stock decrease	0.0	0.0	0.0	0.0	0.0	0.0	0.0	0.0	0.0	0.0	0.0	0.0	0.0	0.0	0.0	0.0
	Loss	1.4	1.3	1.4	1.4	1.7	2.2	2.1	2.4	3.0	4.7	3.7	3.2	3.6	2.6	4.4	4.4
	Non-energy use	12.4	13.6	14.3	16.5	17.6	19.0	17.1	20.0	22.5	21.8	16.9	21.4	26.5	24.7	26.3	21.0
	Natural gas total	38.2	44.1	48.1	53.8	65.1	79.1	103.2	130.4	151.1	167.6	185.6	227.4	250.0	288.1	314.0	332.5
Process	Cement	173.5	192.1	210.7	250.5	281.0	310.6	359.4	395.6	406.8	477.7	546.9	610.0	634.7	702.1	724.2	685.6
Total reference CO_2_ emissions		3214.1	3364.4	3605.0	4249.7	4894.7	5508.8	6111.1	6624.1	6904.7	7567.6	8425.0	9274.6	9859.0	10144.6	10025.5	9779.5
Total Sectoral CO_2_ emissions		3003.4	3250.1	3472.1	4085.6	4680.4	5401.1	6008.7	6546.3	6761.0	7333.7	7904.5	8741.6	9080.5	9534.2	9438.4	9265.1

**Table 6:** Sectoral approach CO_2_ emission inventory of China’s provinces, 1997–2015 (in million tonnes

**Provinces**	**1997**	**1998**	**1999**	**2000**	**2001**	**2002**	**2003**	**2004**	**2005**	**2006**	**2007**	**2008**	**2009**	**2010**	**2011**	**2012**	**2013**	**2014**	**2015**
Beijing	61.9	63.3	66.6	68.2	77.4	77.9	82.0	88.1	92.1	96.7	102.9	99.2	100.4	103.0	94.4	97.2	93.4	92.5	95.2
Tianjin	51.4	51.8	53.0	58.2	60.4	65.1	66.2	78.3	89.0	95.5	103.4	110.2	122.3	136.6	152.0	158.0	157.0	155.4	151.9
Hebei	212.1	211.3	222.8	237.3	251.5	284.4	328.6	374.2	459.1	486.7	529.0	554.4	577.8	647.0	724.6	714.5	768.9	751.9	734.1
Shanxi	148.7	147.5	145.6	148.0	184.2	221.4	251.7	267.9	290.0	320.1	344.9	370.9	375.4	406.5	438.8	466.0	488.2	475.7	440.2
InnerMongolia	97.0	92.4	97.3	105.5	115.8	127.1	149.3	207.3	240.5	290.7	340.0	411.4	445.3	477.4	598.2	621.6	576.2	582.2	584.7
Liaoning	200.7	190.6	184.5	213.0	205.5	220.7	236.7	250.2	279.6	317.9	362.2	371.4	406.9	446.3	455.1	461.0	482.0	484.5	472.1
Jilin	98.6	83.3	84.0	83.0	89.1	92.2	134.3	112.3	143.4	158.9	170.5	179.4	185.7	202.1	233.9	229.5	222.3	222.6	207.6
Heilongjiang	129.1	124.2	122.1	125.3	121.1	117.9	129.6	140.2	158.1	179.0	187.4	197.3	203.2	218.3	247.4	269.2	256.8	269.1	265.5
Shanghai	103.2	104.4	115.6	118.0	122.5	128.4	137.0	148.7	158.9	165.2	174.8	178.2	179.1	187.1	200.2	194.8	201.2	187.7	188.6
Jiangsu	183.9	186.6	194.0	199.4	191.6	220.1	250.8	311.6	396.1	440.9	468.7	496.6	515.6	580.3	633.3	656.2	694.3	704.5	759.5
Zhejiang	115.4	114.9	121.1	131.4	142.9	156.5	178.4	218.0	255.8	290.3	325.3	330.2	338.5	358.6	379.4	377.2	379.0	375.3	375.4
Anhui	109.5	105.8	113.2	118.7	127.0	112.8	168.0	153.8	156.7	175.8	197.7	225.0	251.5	261.9	291.3	318.2	343.1	350.4	351.3
Fujian	44.2	46.6	60.2	56.4	56.4	67.3	82.8	100.2	123.9	135.4	161.3	165.7	187.8	199.4	236.9	232.2	229.4	243.4	230.4
Jiangxi	51.8	50.7	51.4	53.3	58.0	63.3	75.9	89.2	96.4	108.8	127.2	130.3	142.2	148.4	164.0	164.0	197.4	202.3	210.4
Shandong	199.3	212.7	213.5	194.8	232.7	258.8	325.0	397.7	556.5	605.5	663.0	697.7	717.9	766.6	800.8	842.2	761.6	790.4	824.4
Henan	154.3	155.8	157.2	162.1	181.3	194.3	215.7	277.6	336.2	379.0	426.2	435.6	450.7	504.7	548.5	520.7	483.9	535.4	517.8
Hubei	133.9	132.6	135.0	136.8	135.2	154.2	165.2	182.2	189.3	225.2	249.8	254.0	275.2	324.3	373.6	367.6	309.2	310.2	308.2
Hunan	98.0	99.2	80.6	77.3	85.4	92.1	104.9	125.3	178.5	203.2	223.3	226.5	239.0	254.9	285.5	281.7	271.2	269.9	289.2
Guangdong	165.1	172.8	185.2	199.6	210.0	231.5	261.7	296.9	341.8	376.2	410.1	416.9	437.6	471.5	520.6	504.7	496.8	503.8	444.1
Guangxi	50.8	51.8	52.4	56.2	56.1	56.5	66.7	86.6	98.9	112.8	128.2	133.0	151.8	171.8	192.3	204.9	210.0	207.8	269.7
Hainan	7.2	10.4	8.1	8.7	9.2	N/A	15.6	17.2	16.5	19.2	21.7	24.8	27.0	28.9	34.9	37.3	39.5	40.7	42.3
Chongqing	55.4	63.7	69.1	71.3	65.2	70.0	68.4	68.3	81.6	90.0	99.2	126.3	133.1	141.5	160.3	164.8	140.3	156.2	179.3
Sichuan	123.1	123.0	109.3	105.0	108.8	124.0	157.2	175.0	170.1	189.8	208.5	230.1	263.1	303.8	303.4	330.7	343.1	341.3	322.8
Guizhou	72.2	78.3	77.2	81.2	82.9	86.3	110.1	128.6	145.6	169.6	172.0	163.4	184.7	191.5	211.0	230.1	233.2	231.0	233.6
Yunnan	58.0	56.8	54.9	53.0	61.4	72.3	88.8	58.7	133.1	150.2	161.2	163.8	187.2	194.2	205.4	211.7	206.2	194.6	175.9
Shaanxi	68.9	65.7	59.3	58.9	68.4	76.3	86.3	107.7	122.3	128.7	148.1	165.4	186.0	218.6	243.8	261.9	265.6	277.2	276.9
Gansu	50.3	50.9	51.5	54.3	56.1	59.0	67.0	77.9	84.2	89.4	97.8	103.3	100.8	126.5	138.9	152.7	159.6	163.5	158.5
Qinghai	11.5	11.6	13.7	12.1	14.7	15.7	17.6	19.0	19.9	24.4	26.0	31.6	33.5	31.8	36.6	44.6	47.9	48.5	51.1
Ningxia	17.1	17.6	17.4	N/A	N/A	N/A	55.3	65.8	51.7	58.8	66.5	75.4	80.0	95.3	137.3	135.0	142.9	142.6	140.8
Xinjiang	63.2	63.5	62.3	65.4	53.5	69.7	77.2	90.2	101.1	113.9	125.3	137.2	156.7	167.6	203.0	251.5	292.7	329.2	342.5

**Table 7:** Reference approach CO_2_ emission inventory of China’s provinces, 1997–2015 (in million tonnes)

**Provinces**	**1997**	**1998**	**1999**	**2000**	**2001**	**2002**	**2003**	**2004**	**2005**	**2006**	**2007**	**2008**	**2009**	**2010**	**2011**	**2012**	**2013**	**2014**	**2015**
Beijing	60.5	60.3	60.7	63.4	61.7	64.3	69.3	53.9	95.3	81.2	80.5	92.1	107.8	96.7	94.7	96.0	86.8	89.0	83.3
Tianjin	56.7	55.6	57.0	67.1	66.8	68.9	71.4	78.9	89.5	90.3	89.9	92.7	156.4	134.2	148.9	143.1	150.9	150.0	82.4
Hebei	248.9	247.1	251.8	257.7	266.6	290.2	320.8	365.4	409.0	409.3	454.1	482.0	563.4	569.4	623.3	642.4	657.7	624.7	639.4
Shanxi	231.0	232.5	142.0	87.9	93.3	207.7	308.4	322.9	296.5	313.9	238.1	593.7	589.8	654.1	766.2	854.8	1499.0	1553.8	1474.5
Inner mongolia	107.3	100.5	103.4	110.9	117.6	132.1	126.2	209.9	246.4	278.0	328.7	437.7	477.1	562.5	740.5	788.8	783.7	903.2	858.8
Liaoning	243.8	237.1	248.6	290.5	272.4	294.1	319.5	355.8	398.0	417.2	410.7	436.4	457.0	494.7	524.7	543.3	529.9	521.0	502.4
Jilin	108.6	94.4	95.3	95.9	102.5	105.1	116.4	123.6	146.9	164.0	162.0	190.7	202.0	226.1	264.6	265.2	237.4	234.9	218.7
Heilongjiang	184.5	171.0	165.3	172.1	163.2	164.1	171.8	193.3	228.1	231.5	222.6	239.3	274.4	351.7	381.2	393.1	363.4	350.4	347.7
Shanghai	86.6	87.0	92.6	100.8	106.0	109.1	124.0	134.1	142.4	138.4	131.8	144.3	142.4	161.3	170.5	168.4	181.6	156.7	161.8
Jiangsu	202.8	202.5	207.9	216.4	217.0	232.3	268.0	314.1	386.8	419.5	436.3	462.2	485.9	546.2	613.1	620.1	638.4	621.1	634.2
Zhejiang	116.6	108.8	120.7	100.2	147.8	163.0	184.0	231.8	267.4	302.1	326.2	338.6	351.8	375.9	398.5	383.6	387.7	380.9	381.6
Anhui	115.6	112.6	114.8	122.4	129.0	133.2	150.4	165.4	171.6	188.7	197.5	240.4	273.3	283.0	315.4	355.9	387.2	401.7	392.8
Fujian	41.8	43.2	49.3	53.9	53.6	61.9	75.0	86.7	99.4	112.6	128.7	137.0	161.9	179.6	210.2	208.5	203.5	229.2	234.4
Jiangxi	56.9	51.5	47.8	51.6	52.7	56.3	67.8	83.4	90.3	101.0	107.3	115.5	117.3	134.4	144.7	146.4	162.2	165.5	170.4
Shandong	264.7	261.3	260.6	261.5	303.3	345.9	424.2	518.3	661.9	745.4	765.6	831.3	880.1	929.1	976.5	1007.7	944.4	998.0	1052.2
Henan	176.6	133.5	139.6	140.3	156.5	162.7	231.9	239.3	343.4	352.5	394.8	330.9	472.0	573.4	654.1	545.8	594.4	557.8	537.2
Hubei	121.8	110.6	123.4	127.8	126.9	135.1	150.5	169.0	167.7	199.7	210.5	212.1	232.8	279.6	321.9	311.4	252.9	251.8	252.7
Hunan	74.7	101.2	79.0	75.9	82.0	94.5	104.2	114.4	167.3	184.9	202.4	198.3	212.0	231.7	269.7	275.3	266.4	255.9	250.5
Guangdong	155.5	146.7	157.8	182.1	185.5	198.7	225.6	251.4	272.4	310.4	327.6	357.1	394.8	445.0	501.2	486.6	493.0	541.6	506.7
Guangxi	44.8	44.0	44.8	47.4	44.9	41.7	49.6	67.2	70.5	80.9	89.6	95.8	109.6	134.2	173.3	192.8	189.6	183.4	173.2
Hainan	3.0	9.4	3.2	4.8	4.9	N/A	8.3	5.8	8.1	14.8	34.5	35.7	38.3	44.9	53.0	54.3	52.1	58.9	65.3
Chongqing	57.0	54.9	60.3	60.6	56.5	58.0	54.5	64.5	73.4	83.9	91.5	119.9	136.4	138.3	150.7	149.4	134.1	144.8	146.6
Sichuan	119.8	117.8	99.9	104.1	105.8	113.7	148.7	169.8	159.3	167.7	207.7	234.0	271.1	283.8	280.5	289.6	284.8	360.0	307.8
Guizhou	100.2	105.1	62.4	61.4	55.6	68.6	111.8	123.4	144.1	167.5	159.6	195.3	227.2	246.9	268.7	287.2	314.3	319.5	327.6
Yunnan	64.1	61.1	57.9	53.6	58.6	63.3	82.0	59.7	124.5	140.7	137.4	150.4	165.5	176.3	187.8	195.4	195.8	187.7	178.7
Shaanxi	78.3	77.8	73.8	68.7	70.4	84.9	93.5	116.3	217.5	187.3	234.2	274.8	262.3	308.3	344.5	405.1	482.9	639.0	665.8
Gansu	65.1	66.7	66.6	70.7	73.1	79.1	90.2	99.5	104.4	110.3	118.0	126.5	124.4	145.1	169.7	173.8	181.5	180.4	176.6
Qinghai	12.2	12.3	14.5	12.5	15.4	16.5	18.3	19.7	21.2	24.3	25.1	32.2	35.4	37.2	50.2	58.6	70.7	74.5	51.3
Ningxia	24.7	29.4	28.2	N/A	N/A	N/A	38.0	61.4	74.0	82.4	98.3	109.5	140.1	151.5	191.0	188.6	187.8	195.2	193.4
Xinjiang	85.1	89.3	86.7	89.4	93.6	92.1	103.0	121.0	132.4	148.5	152.4	179.0	214.4	240.9	286.2	333.4	336.4	542.3	535.0
Total	3309.2	3225.2	3115.9	3152.4	3284.1	3639.2	4307.3	4919.9	5809.7	6248.9	6563.6	7485.4	8276.9	9136.0	10275.5	10564.6	11250.5	11872.9	11603.0

**Table 8:** Emission factors by different sources and CV

	**IPCC**	**NBS**	**NDRC**	**NC1994**	**NC2005**	**MEIC**	**UN-China**	**UN average**	**Liu** ***et al*** **.'s nature**	**Average value**	**CV**
Net caloric value (*PJ/*10_4_ *tonnes*, 10_8_*m*_3_)											
Raw Coal	0.28	0.21	0.21	0.21	0.22	0.19	0.21	0.29	0.21	0.23	15%
Cleaned Coal	0.27	0.26	0.23	0.24	0.23	0.26	0.21	0.29	0.26	0.25	9%
Other Washed Coal	0.27	0.15	0.23	0.21	0.23	0.15	0.21	0.29	0.15	0.21	23%
Briquettes	0.26	0.18	0.17	0.20	0.17	0.18	0.21	0.29	0.18	0.20	20%
Coke	0.28	0.28	0.28	0.28	0.28	0.28	0.26	0.26	0.28	0.28	3%
Coke Oven Gas	1.88	1.63	1.74	1.63	1.74	1.67	1.88	1.88	1.61	1.74	6%
Other Gas	1.88	0.84	1.58	0.84	1.58	0.52	1.88	1.88	0.83	1.31	39%
Other Coking Products	0.43	0.28	0.28	0.28	0.28	0.42	0.43	0.43	0.28	0.35	21%
Crude Oil	0.42	0.42	0.43	0.42	0.43	0.42	0.42	0.42	0.43	0.42	1%
Gasoline	0.44	0.43	0.45	0.45	0.45	0.43	0.45	0.45	0.44	0.44	2%
Kerosene	0.44	0.43	0.45	0.45	0.45	0.43	0.43	0.43	0.44	0.44	2%
Diesel Oil	0.43	0.43	0.43	0.45	0.43	0.43	0.42	0.42	0.43	0.43	2%
Fuel Oil	0.40	0.42	0.40	0.40	0.40	0.42	0.40	0.40	0.43	0.41	2%
LPG	0.47	0.50	0.47	0.47	0.47	0.50	0.46	0.46	0.51	0.48	4%
Refinery Gas	0.50	0.46	0.46	0.40	0.46	0.46	0.42	0.42	0.47	0.45	6%
Other Petroleum Products	0.40	0.42	0.45	0.40	0.45	0.42	0.42	0.42	0.43	0.42	4%
Natural Gas	3.44	3.89	3.89	3.90	3.89	3.89	3.44	3.44	3.89	3.74	6%
Carbon content (*tonne C/TJ*)											
Raw Coal	25.80	26.37	26.37	24.26	25.83	25.80	25.80	25.80	26.32	25.82	2%
Cleaned Coal	26.80	25.41	25.41	26.35	27.82	25.80	26.80	26.80	26.32	26.39	3%
Other Washed Coal	26.80	25.41	25.41	24.26	27.82	25.80	26.80	26.80	26.32	26.16	4%
Briquettes	25.80	33.56	33.56	24.26	33.56	25.80	25.80	25.80	26.32	28.27	13%
Coke	29.20	29.42	29.42	29.50	28.84	25.52	29.20	29.20	31.38	29.08	5%
Coke Oven Gas	12.10	13.58	13.58	20.00	14.00	15.16	12.10	12.10	21.49	14.90	22%
Other Gas	12.10	12.20	12.20	12.10	12.20	15.16	12.10	12.10	21.49	13.52	22%
Other Coking Products	25.80	29.50	29.50	25.80	20.00	19.91	25.80	25.80	27.45	25.51	13%
Crude Oil	20.00	20.08	20.08	20.00	20.08	19.91	20.00	20.00	20.08	20.03	0%
Gasoline	18.90	18.90	18.90	18.90	18.90	19.91	18.90	18.90	18.90	19.01	2%
Kerosene	19.50	19.60	19.60	19.60	19.60	19.91	19.50	19.50	19.60	19.60	1%
Diesel Oil	20.20	20.20	20.20	20.20	20.20	19.91	20.20	20.20	20.20	20.17	0%
Fuel Oil	21.10	21.10	21.10	21.10	21.10	19.91	21.10	21.10	21.10	20.97	2%
LPG	17.20	17.20	17.20	17.20	17.20	19.91	17.20	17.20	17.20	17.50	5%
Refinery Gas	15.70	18.20	18.20	15.70	18.20	19.91	15.70	15.70	20.00	17.48	10%
Other Petroleum Products	20.00	20.00	20.00	20.00	20.00	19.91	20.00	20.00	20.20	20.01	0%
Natural Gas	15.30	15.32	15.32	15.30	15.32	15.16	15.30	15.30	15.32	15.29	0%
Oxygenation efficiency (*tonne* CO_2_*/ton*)											
Raw Coal	0.98	0.94	0.94	0.90	0.92	1.00	1.00	1.00	0.92	0.95	4%
Cleaned Coal	0.98	0.98	0.98	0.90	0.92	1.00	1.00	1.00	0.92	0.96	4%
Other Washed Coal	0.98	0.98	0.98	0.90	0.92	1.00	1.00	1.00	0.92	0.96	4%
Briquettes	0.98	0.90	0.90	0.90	0.90	1.00	1.00	1.00	0.92	0.94	5%
Coke	0.98	0.93	0.93	0.97	0.93	1.00	1.00	1.00	0.92	0.96	3%
Coke Oven Gas	0.99	0.99	0.99	0.99	0.99	1.00	1.00	1.00	0.92	0.99	2%
Other Gas	0.99	0.99	0.99	0.99	0.99	1.00	1.00	1.00	0.92	0.99	2%
Other Coking Products	0.99	0.93	0.93	0.97	0.93	1.00	1.00	1.00	0.92	0.96	3%
Crude Oil	0.99	0.98	0.98	0.98	0.98	1.00	1.00	1.00	0.98	0.99	1%
Gasoline	0.99	0.98	0.98	0.98	0.98	1.00	1.00	1.00	0.98	0.99	1%
Kerosene	0.99	0.98	0.98	0.98	0.98	1.00	1.00	1.00	0.98	0.99	1%
Diesel Oil	0.99	0.98	0.98	0.98	0.98	1.00	1.00	1.00	0.98	0.99	1%
Fuel Oil	0.99	0.98	0.98	0.98	0.98	1.00	1.00	1.00	0.98	0.99	1%
LPG	0.99	0.99	0.99	0.99	0.99	1.00	1.00	1.00	0.98	0.99	1%
Refinery Gas	0.99	0.99	0.99	0.99	0.99	1.00	1.00	1.00	0.98	0.99	1%
Other Petroleum Products	0.99	0.98	0.98	0.98	0.98	1.00	1.00	1.00	0.98	0.99	1%
Natural Gas	0.99	0.99	0.99	0.99	0.99	1.00	1.00	1.00	0.99	0.99	0%
Emission factor											
Raw Coal	2.61	1.90	1.90	1.67	1.94	1.80	1.98	2.77	1.83	2.05	18%
Cleaned Coal	2.57	2.41	2.12	2.13	2.17	2.49	2.05	2.88	2.31	2.35	11%
Other Washed Coal	2.57	1.41	2.12	1.66	2.17	1.46	2.05	2.88	1.33	1.96	26%
Briquettes	2.39	1.97	1.93	1.60	1.93	1.68	1.98	2.77	1.60	1.98	18%
Coke	2.96	2.85	2.85	2.99	2.79	2.66	2.82	2.82	2.96	2.86	3%
Coke Oven Gas	8.26	8.04	8.55	11.84	8.85	9.30	8.34	8.34	11.67	9.24	15%
Other Gas	8.26	3.73	6.98	3.70	6.98	2.91	8.34	8.34	6.02	6.14	33%
Other Coking Products	4.03	2.86	2.86	2.61	1.94	3.05	4.07	4.07	2.59	3.12	23%
Crude Oil	3.07	3.02	3.08	3.01	3.08	3.05	3.10	3.10	3.10	3.07	1%
Gasoline	3.05	2.93	3.04	3.04	3.04	3.14	3.11	3.11	2.99	3.05	2%
Kerosene	3.10	3.04	3.15	3.15	3.15	3.14	3.09	3.09	3.10	3.11	1%
Diesel Oil	3.15	3.10	3.15	3.25	3.15	3.11	3.15	3.15	3.12	3.15	1%
Fuel Oil	3.09	3.17	3.05	3.05	3.05	3.05	3.13	3.13	3.26	3.11	0.02
LPG	2.95	3.13	2.95	2.95	2.95	3.66	2.87	2.87	3.15	3.06	8%
Refinery Gas	2.82	3.04	3.04	2.29	3.04	3.36	2.41	2.41	3.38	2.87	14%
Other Petroleum Products	2.90	3.01	3.24	2.89	3.24	3.05	3.12	3.12	3.12	3.08	4%
Natural Gas	1.91	2.17	2.17	2.17	2.17	2.16	1.93	1.93	2.16	2.08	5%

**Table 9:** Uncertainties in China’s CO_2_ emission inventories (97.5% confidence level)

	**2015**			**2014**			**2013**		
Total	8,579	−16%	25%	8,714	−16%	25%	8,832	−17%	26%
Primary and residence	502	−24%	7%	471	−24%	8%	448	−25%	9%
Coal mining and dressing	80	−17%	44%	104	−18%	45%	133	−20%	47%
Smelting and pressing of ferrous metal	1,690	−20%	18%	1,803	−20%	18%	1,762	−20%	18%
Non-metal Mineral Products	588	−15%	45%	630	−16%	44%	612	−18%	44%
Electricity generation	3,837	−22%	43%	3,896	−22%	44%	4,062	−22%	44%
Other industry	893	−21%	17%	878	−21%	17%	904	−21%	19%
Construction	47	−8%	16%	45	−9%	18%	43	−9%	16%
Service	942	−9%	1%	887	−9%	2%	867	−8%	3%
	2012			2011			2010		
Total	8,446	−17%	27%	8,132	−16%	27%	7,358	−16%	27%
Primary and residence	420.75	−24%	11%	403.22	−24%	12%	379.41	−23%	14%
Coal mining and dressing	125.02	−21%	47%	121.02	−21%	48%	114.71	−22%	49%
Smelting and pressing of ferrous metal	1,672	−19%	17%	1,599	−19%	18%	1,462	−19%	17%
Non-metal Mineral Products	615.69	−17%	45%	634.67	−17%	45%	576.41	−17%	46%
Electricity generation	3,855	−22%	45%	3,632	−22%	45%	3,196	−22%	45%
Other industry	905.63	−20%	21%	956.52	−20%	22%	909.6	−18%	23%
Construction	38.733	−9%	17%	39.286	−9%	17%	37.01	−9%	17%
Service	813.74	−8%	3%	746.06	−8%	3%	682.79	−7%	4%
	2009			2008			2007		
Total	6,856	−16%	28%	6,354	−16%	28%	6,151	−16%	28%
Primary and residence	352.88	−22%	17%	343.75	−22%	18%	367.35	−19%	20%
Coal mining and dressing	110.29	−21%	50%	70.49	−19%	46%	68.096	−20%	47%
Smelting and pressing of ferrous metal	1,386	−19%	17%	1,198	−19%	17%	1,138	−19%	17%
Non-metal Mineral Products	560.49	−18%	46%	534.99	−19%	44%	507.9	−18%	46%
Electricity generation	2,900	−22%	45%	2,686	−22%	46%	2,601	−22%	45%
Other industry	884.65	−18%	24%	873.16	−19%	23%	849.16	−19%	23%
Construction	32.413	−8%	18%	29.566	−9%	18%	31.195	−9%	17%
Service	629.68	−8%	5%	618.38	−7%	5%	587.95	−7%	6%
	2006			2005			2004		
Total	5,649	−16%	28%	5,091	−16%	28%	4,399	−16%	29%
Primary and residence	381.73	−16%	21%	318.16	−18%	26%	300.39	−17%	27%
Coal mining and dressing	59.381	−20%	47%	61.406	−17%	40%	31.595	−16%	45%
Smelting and pressing of ferrous metal	996.18	−19%	16%	886.69	−19%	15%	630.23	−19%	15%
Non-metal Mineral Products	459.55	−18%	46%	438.82	−17%	49%	286.65	−13%	44%
Electricity generation	2,431	−22%	46%	2,156	−22%	46%	1,891	−22%	45%
Other industry	768.58	−19%	23%	695.59	−21%	22%	763.06	−20%	30%
Construction	30.908	−10%	18%	28.41	−10%	18%	26.009	−11%	19%
Service	521.71	−7%	7%	505.3	−6%	7%	470.63	−6%	7%
	2003			2002			2001		
Total	3,835	−16%	29%	3,261	−16%	29%	3,058	−15%	29%
Primary and residence	283.33	−16%	27%	238.64	−18%	29%	271.11	−19%	30%
Coal mining and dressing	64.628	−28%	30%	46.873	−19%	46%	46.479	−18%	45%
Smelting and pressing of ferrous metal	578.84	−17%	16%	451.72	−18%	17%	430.91	−18%	17%
Non-metal Mineral Products	284.96	−18%	47%	226.55	−15%	45%	226.6	−15%	45%
Electricity generation	1,730	−22%	46%	1,454	−22%	45%	1,302	−22%	45%
Other industry	490.39	−17%	19%	476.41	−18%	18%	440.09	−18%	18%
Construction	22.212	−11%	21%	21.136	−11%	21%	20.454	−11%	21%
Service	380.98	−6%	8%	346.4	−6%	8%	320.5	−5%	9%
	2000								
Total	2,830	−15%	29%						
Primary and residence	216.68	−17%	33%						
Coal mining and dressing	43.759	−18%	44%						
Smelting and pressing of ferrous metal	390.58	−17%	16%						
Non-metal Mineral Products	199.94	−15%	44%						
Electricity generation	1,218	−22%	45%						
Other industry	427.08	−18%	19%						
Construction	20.288	−12%	23%						
Service	314.07	−5%	8%						
The first column of each year refers to the emission estimates, in million tonnes. The percentages followed indicate the 97.5% Confidence Interval around the central estimate.									
