# Supplementary material for: China CO2 emission accounts 1997–2015
Source: Sci Data. 2018 Jan 16;5:170201. doi: 10.1038/sdata.2017.201 (PMC5769543; doi:10.1038/sdata.2017.201)
Supplement: Supplementary Information [file sdata2017201-s2.docx]

**Supplementary Information of**

# China CO_2_ emission accounts 1997-2015

Yuli Shan^1^, Dabo Guan^1,2,*^, Heran Zheng^1^, Jiamin Ou^1^, Yuan Li^1,*^, Jing Meng^3^, Zhifu Mi^1,4^, Zhu Liu^1,*^ and Qiang Zhang^2^

1. Water Security Research Centre, School of International Development, University of East Anglia, Norwich NR4 7TJ, UK

2. Department of Earth System Sciences, Tsinghua University, Beijing, 100080, China.

3. Department of Politics and International Studies, University of Cambridge, Cambridge CB3 9DT, UK

4. Bartlett School of Construction and Project Management, University College London, London WC1E 7HB, UK

Correspondence emails: [dabo.guan@uea.ac.uk](mailto:dabo.guan@uea.ac.uk), [y.li4@uea.ac.uk](mailto:y.li4@uea.ac.uk), [zhu.liu@uea.ac.uk](mailto:zhu.liu@uea.ac.uk)

**MATLAB code**

We take Anhui 2007 as an example to show the calculation of emission inventory with MATLAB R2014a using the energy data.

*% Read emission factors and energy data from the excel files*

NCV=xlsread('Emission factors', 'NCV', 'A2:Q2'); *%NCV_i_ refers to Table 1*

NCV=repmat(NCV,68,1);

CC=xlsread('Emission factors', 'CC', 'A2:Q2'); *%CC_i_ refers to Table 1*

CC=repmat(CC,47,1);

O = xlsread('Emission factors', 'Oxygenation Efficiency', 'B2:R48'); *%O_ij_ refers to Table 3*

Energy=xlsread('Province Energy inventory 2007','Anhui','B3:R70');

*% Convert physical energy consumption to calories*

E_PJ1 = Energy .* NCV;

E_PJ1(1,:) = sum(E_PJ1(2:48,:));

E_PJ2 = E_PJ1;

*% Remove non-energy use from the total consumption*

E_NE = zeros (68,17);

E_NE (23:27, 1:17) = repmat (E_PJ1(65,:),5,1) .* E_PJ2(23:27,:) ./ repmat (sum(E_PJ1(23:27,:)),5,1);

E_NE (:,16) = E_PJ2 (:,16);

E_NE(isnan(E_NE))=0;

*% Include energy combustion consumption during transformation process*

E_Trans = zeros (68,17);

E_Trans (40,:) = E_PJ1(52,:)+E_PJ1(53,:);

E_Trans (44,10:13) = E_PJ1(67,10:13)+E_PJ1(68,10:13);

E = E_PJ2(1:48,:)-E_NE(1:48,:)+E_Trans(1:48,:);

E(1,:) = sum(E(2:48,:));

*% Calculate the energy-related emissions*

CO2_E = E (2:48,:) .* CC .* O / 100;

CO2_E(CO2_E<0)=0;

*% Calculate the process-related emissions*

EF_cement=0.2906;

Prod_cement=54.0223; *%Prod_cement here refers to Anhui’s cement production in 2007*

CO2_cement = Prod_cement .* EF_cement;

*% Construct the emission inventory*

CO2 = zeros (48,19);

CO2 (2:48,1:17) = CO2_E;

CO2 (28,18)= CO2_cement;

CO2 (:, 19) = sum(CO2,2);

CO2 (1,:) = sum(CO2(2:48,:))
